# The influence of clustering coefficient on word-learning: how groups of similar sounding words facilitate acquisition

**DOI:** 10.3389/fpsyg.2014.01307

**Published:** 2014-11-18

**Authors:** Rutherford Goldstein, Michael S. Vitevitch

**Affiliations:** Department of Psychology, University of KansasLawrence, KS, USA

**Keywords:** network science, word-learning, neighborhood density, clustering coefficient

## Abstract

Clustering coefficient, *C*, measures the extent to which neighbors of a word are also neighbors of each other, and has been shown to influence speech production, speech perception, and several memory-related processes. In this study we examined how *C* influences word-learning. Participants were trained over three sessions at 1-week intervals, and tested with a picture-naming task on nonword-nonobject pairs. We found an advantage for novel words with high *C* (the neighbors of this novel word are likely to be neighbors with each other), but only after the 1-week retention period with no additional exposures to the stimuli. The results are consistent with the spreading-activation network-model of the lexicon proposed by Chan and Vitevitch (2009). The influence of *C* on various language-related processes suggests that characteristics of the individual word are not the only things that influence processing; rather, lexical processing may also be influenced by the relationships that exist among words in the lexicon.

## Introduction

Network science has been used to examine various aspects of the lexicon including semantic relationships among words (Hills et al., 2010), phonological relationships among words in various languages (Arbesman et al., 2010), the acquisition of words in typically developing children (Carlson et al., 2011), and the acquisition of words in children with language delays (Beckage et al., 2011). When used in conjunction with conventional psycholinguistic tasks, network science enables researchers to examine how structural relationships in the lexicon influence language processing.

**Figure 1:**
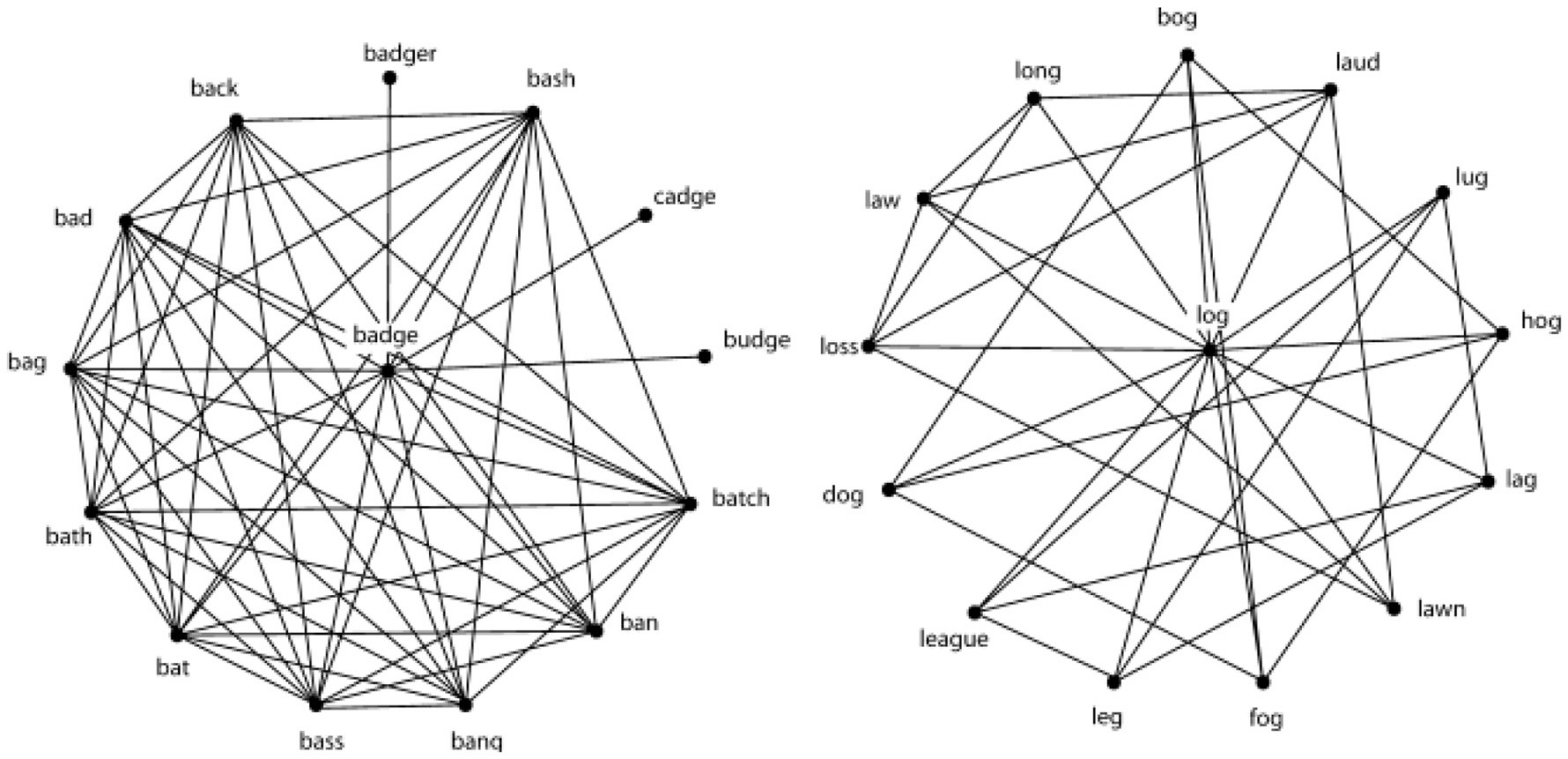
**The word BADGE has many connections within the neighborhood, thus a high clustering coefficient**. The word LOG has few connections within the neighborhood, thus a low clustering coefficient.

Consider the measure known as clustering coefficient, *C* (Watts and Strogatz, 1998). In a network of phonologically related words (Vitevitch, 2008), *C* assesses the extent to which neighbors of a word are also neighbors of each other. Clustering coefficient should not be confused with neighborhood density (Luce and Pisoni, 1998); they are different measures, and, as shown in Chan and Vitevitch (2009) are not correlated with each other. To illustrate the difference, consider the words *badge* and *log* in Figure 1, which have the same number of phonological neighbors. However, many neighbors of the word *badge* are also neighbors with each other. Although some neighbors of *log* are neighbors with each other there are fewer such connections among the neighbors of *log* (which has low *C*) than there are among the neighbors of *badge* (which has high *C*). It is this relationship among the neighbors of a word that is assessed by *C*. (See the Method for a more precise definition of *C*.).

Chan and Vitevitch (2009) and see also Yates (2013) found that low *C* words were recognized more quickly and accurately than high *C* words. Furthermore, it was found that low *C* words were produced more quickly in a speeded picture-naming task and more accurately in an analysis of speech errors than high *C* words (Chan and Vitevitch, 2010). Thus, a very clear picture is emerging for the influence of *C* on the retrieval of known words with *well-established representations* in the lexicon: low *C* words are retrieved more quickly and accurately than high *C* words.

Chan and Vitevitch (2009) accounted for the influence of *C* on lexical retrieval with a model that proposed that activation diffused across the lexical network. For low *C* words (*log* in Figure 1), Chan and Vitevitch suggested that the small number of interconnections among the neighbors results in some of the activation from the neighbors spreading back to the target word, and the remaining activation dispersing to the rest of the network (i.e., words related to the neighbors of *log*, but not shown in Figure 1). The strongly activated target word, *log*, would “stand out” from the less activated neighbors (and less activated neighbors of neighbors), resulting in rapid and accurate retrieval of words with low *C*.

For high *C* words (*badge* in Figure 1), where the neighbors are highly interconnected with each other, most of the activation remains amongst the interconnected neighbors rather than spread back to the target word or to the rest of the network as happens for low *C* words. With a highly activated target word as well as highly activated neighbors, discrimination of the target word becomes more difficult, resulting in slower and less accurate retrieval of high *C* words. Note that for high *C* words, activation will spread from the target word to the rest of the lexicon, but to a lesser extent than for low *C* words. Vitevitch et al. (2011) confirmed via computer simulation the model proposed by Chan and Vitevitch (2009). That simulation not only accounted for the influences of *C* observed by Chan and Vitevitch, but also accounted for the independent and well-studied influence of neighborhood density on spoken word recognition (Luce and Pisoni, 1998), further clarifying the influence of these variables on the retrieval of known-words with well-established representations in the lexicon.

However, for lexical items with *partially degraded representations* being retrieved from short-term memory, a different influence of *C* on processing is observed. Vitevitch et al. (2012) found in a serial recall task—used to examine the process of redintegration where lexical representations in long-term memory activate degraded representations in short-term memory—an advantage for high *C* words over low *C* words. Vitevitch et al. (2012) again appealed to the differential amount of activation that circulates amongst phonological neighbors with either high or low *C* to account for this influence on the retrieval of partially degraded representations of lexical items. For high *C* words, the recirculation of activation amongst the neighbors continually activates a small set of phonologically similar representations in long term memory, which are used to partially activate the degraded representation of the lexical item in short-term memory (for more about redintegration see Hulme et al., 1997). For low *C* words, the dispersion of activation to the rest of the network partially activates many and varied lexical representations, which provides little (consistent) support to the decaying representation of the lexical item in short term memory. The difference in the amount of activation available to support the redintegration of degraded lexical representations of words with high vs. low *C* accounts for the performance on such words observed by Vitevitch et al. (2012) in the serial recall task.

Another situation where one finds “degraded” representations of lexical items—or more precisely, *incomplete representations*—is during the initial acquisition of word-forms (e.g., Gaskell and Dumay, 2003). A model described by Storkel (2011) based on the work of Carpenter and Grossberg (1987) provides some insight into how *C* may influence the word learning process, when newly acquired lexical representations are incomplete. Storkel's model describes a three stage process: triggering, configuration, and engagement. The word-learning process is “triggered” when there is significant mismatch between a newly encountered word and existing lexical representations. The mismatch between the newly encountered word and existing lexical representations indicates that the word is not known, and must therefore be learned (i.e., new lexical and semantic representations must be formed). If there is a match between the encountered word and existing lexical representations (i.e., the word is known), then the corresponding lexical representation is simply updated. That is, the corresponding lexical and semantic level representations will be adjusted to better match the encountered word.

Once learning is triggered, lexical information is stored in long-term memory through the process known as configuration. In the configuration process information in long term memory is either created or modified. If learning has been triggered by encountering a novel word, the lexical and semantic representations will be created in the lexicon. In the case of encountering an already known word, the already existing lexical and semantic information will be updated to better match the encountered word.

Once a lexical representation has been established during the configuration process, lexical engagement must occur. During lexical engagement, the representation will integrate with other existing representations in the lexicon by establishing connections with them. These connections allow the new representation to interact with other representations in the same way that existing representations interact with each other (e.g., neighborhood density effects or *C* effects).

To further examine how *C* influences the retrieval of less well-established, or, more precisely, *nascent*, lexical representations, we used a well-established word-learning paradigm, in which participants are given several blocks of exposure to pairings of nonwords and nonobjects, and tested with a picture-naming task after each block of exposure (Storkel et al., 2006). Based on Chan and Vitevitch (2009), we reasoned that for a novel word with high *C*, the activation that circulates amongst the neighbors would strengthen the nascent lexical representation, allowing it to be better incorporated into the lexicon at the engagement stage of word learning (where connections to other words in the lexicon are established). In contrast, the dispersion of activation to the rest of the network that occurs for low *C* words would—as in the process of redintegration examined in Vitevitch et al. (2012)—provide inadequate support to the nascent lexical representation at the engagement stage of word learning, resulting in an advantage for novel words with high *C* over novel words with low *C* at later stages of testing.

## Methods

### Participants

Thirty-two participants, enrolled in lower level psychology courses at the University of Kansas, took part in the experiment for extra credit. The experiment was approved by the Institutional Review Board at the University of Kansas. None of the participants reported speech or hearing problems or uncorrected-visual disorders.

### Stimuli

The stimuli (see Supplementary Material) consisted of 24 phonotactically legal (in English), monosyllabic nonwords that contained three phonemes with a consonant-vowel-consonant (CVC) structure. Half of the items had high *C* (*mean* = 0.55; *sd* = 0.12) and half had low *C* [*mean* = 0.23; *sd* = 0.08; *t*_(22)_ = 7.57, *p* < 0.0001]. *C* is the ratio of the number of existing connections in a neighborhood compared to the number of possible connections in a neighborhood. A value close to one indicates many connections among the neighbors, whereas a value close to zero indicates few connections among the neighbors. A more precise definition is provided in Equation (1) (Watts and Strogatz, 1998):

(1) $$C_{i}=\frac{2\left|\left\{e_{jk}\right\}\right|}{k_{i}(k_{i}-1)}$$

*e_jk_* refers to the presence of a connection between two neighbors (*j* and *k*) of node *i*, |...| is used in this case to indicate cardinality (i.e., the number of elements in the set, not the absolute value), and *k_i_* refers to the degree (i.e., neighborhood density) of node *i*. Thus, the clustering coefficient is the proportion of connections that exist among the neighbors of a given node divided by the number of connections that could exist among the neighbors of a given node. The *C* value of the nonword stimuli was calculated by assessing the connections among the real word phonological neighbors of each nonword.

There were no significant differences between high and low *C* words on several variables that influence processing (see Table 1): *segment probability, biphone probability*, and *number of real word neighbors*. Segment probability and biphone probability values were obtained from Vitevitch and Luce (2004).

**Table 1:** **Variables controlled in the two groups of nonwords varying in *C***.

**Variable**	**High *C***	**Low *C***
Phonotactic probability	0.12 (0.04)	0.13 (0.04)
Biphone probability	0.005 (0.005)	0.004 (0.004)
Number of real word neighbors	13.25 (4.12)	11.63 (3.36)
Stimulus onset (measured in seconds)	0.008 (0.004)	0.01 (0.01)
Stimulus offset (measured in seconds)	0.009 (0.006)	0.01 (0.009)
Stimulus duration (measured in seconds)	0.51 (0.06)	0.53 (0.10)
File duration (measured in seconds)	0.52 (0.06)	0.56 (0.10)
Concreteness rating	4.44 (0.87)	4.47 (0.86)
First word associate strength	0.15 (0.08)	0.14 (0.07)
Second word associate strength	0.09 (0.03)	0.10 (0.04)
Semantic set size	10.5 (0.52)	10.5 (0.52)

*Mean values are reported (with standard deviations in parentheses). None of the differences between high and low C were statistically significant (all p's > 0.35). Phonotactic probability refers to how often a phoneme occurs in a certain position (Jusczyk et al., 1994), biphone probability refers to how often two phonemes occur next to each other (Jusczyk et al., 1994), number of real word neighbors refers to how many words can be formed by substituting, adding, or deleting a single phoneme, stimulus onset refers to the amount of silence between the beginning of the sound file and the start of the stimulus, stimulus offset refers to the amount of silence between the end of the stimulus and the end of the sound file, stimulus duration refers to the total duration of the stimulus within the sound file, file duration refers to the total duration of the sound file, concreteness rating refers to the extent to which the nonobject resembles an object in the real world (assessed in Kroll and Potter, 1984), first word associate strength refers to the associative strength of the most common semantic associate of a nonobject (assessed in Storkel and Adlof, 2009), second word associate strength refers to the associative strength of the second most common semantic associate of a nonobject (assessed in Storkel and Adlof, 2009), and semantic set size refers to the total number of words semantically associated with a nonobject (assessed in Storkel and Adlof, 2009)*.

Each nonword was randomly paired with a picture of a nonsense-object from Kroll and Potter (1984), henceforth called nonobjects to act as a referent. As shown in Table 1, there were no significant differences between the pictures assigned to high and low *C* words on *concreteness ratings* (degree to which the nonobject resembles an object in the real world; assessed in Kroll and Potter, 1984), first *word associate strength*, second *word associate strength*, and *semantic set size* (assessed in Storkel and Adlof, 2009).

A male native-speaker of American English (the second author) produced all of the nonwords at a normal rate and loudness level in a sound-attenuated booth. Recordings were made using a Marantz PMD671 recorder, and transferred directly to hard-drive for editing using Praat (Boersma and Weenink, 2009). There were no significant differences between the high and low *C* nonwords on various measures of duration; see Table 1.

### Procedure

A common word-learning methodology was used in this experiment (Storkel et al., 2006; Storkel and Lee, 2011). To make learning more manageable for participants in the limited time they were in the laboratory, the 24 stimuli were randomly split into two lists, each containing 6 high *C* and 6 low *C* nonwords. The experiment occurred across three separate sessions, with ~1 week between each session. In session one, participants were trained on the nonword-nonobject pairings in the first list. Nonword-nonobject pairs in each list were presented in a different random order in each training period and for each participant. During training, participants were presented with an image of a nonobject on the screen while the associated nonword was presented auditorily over headphones. The nonwords appeared as the final word in a set of short phrases (e.g., “This is a ____,” “Look at the _____,” “Remember, it's a _____,” “Listen closely, it's called a _____,” “Don't forget the ____”). Each phrase was presented only once, giving the participant 5 exposures to each nonword-nonobject pairing before the next pairing was presented.

Training ended after all 12 nonword-nonobject pairs in the list were presented, and was followed by a test using a picture-naming task. Trials in the picture-naming task proceeded in the following way: ^*****^ appeared for 1000 ms to signal the start of a trial, followed by a nonobject in the center of the screen. Participants were to say out loud the nonword paired with the nonobject. There was no time limit for participants to respond, but they were instructed to answer as quickly and as accurately as possible. Responses triggered a PsyScope button box voice key, which recorded millisecond response latencies. Upon completion of a trial the next trial began. The nonobjects were presented in random order.

After the test, participants took part in a second training period with the same set of nonword-nonobject pairings, followed by another test. This concluded session one. After an interval of ~1 week (*M* = 7.08 days), participants returned to the laboratory for session two. The second session began with a third and final test of list one. Note that participants did not receive any additional exposure to the nonword-nonobject pairings before this final test. Participants were then trained on list 2 of the nonwords-nonobjects, and then tested with the picture-naming task. After the first test, participants were trained on list 2 a second time, and given a second test of list 2. After another interval of ~1 week (*M* = 7.16 days), participants returned for the third and final session of the experiment, where they were given the third and final test of list 2.

## Results

No differences or interactions were observed across lists, so we collapsed across lists in subsequent analyses. Incorrect responses and responses that exceeded two standard deviations above (>9500 ms) or below the mean (<500 ms) were not included in the analyses. Not surprisingly, the long (and highly variable) reaction times in the picture-naming task in the present experiment failed to show any statistically significant differences *F*_(5, 31)_ = 2.16, *p* = 0.06. Due to the significant interaction between *C* and testing period in the accuracy data, *post-hoc* analyses were performed on the individual testing periods for the reaction times, however no significant effects of *C* were observed at Test 1 (High *C*: *Mean* = 4527 ms, *SD* = 5473 ms; Low *C*: *Mean* = 3250 ms *SD* = 2284 ms), at Test 2 (High *C*: *Mean* = 2887 ms, *SD* = 1737; Low *C*: *Mean* = 2409 ms, *SD* = 1300 ms), or at Test 3 (High *C*: *Mean* = 3409 ms, *SD* = 2107 ms; Low *C*: *Mean* = 2864 ms, *SD* = 1537 ms) (all *p* > 0.1).

It is not uncommon to see long (Test 1 mean response time ~4 s, Test 2 mean response time ~3 s, and Test 3 mean response time ~3 s) and highly variable reaction times in word-learning tasks like that used in the present study. Furthermore, when these response times are compared to the picture-naming task used in Chan and Vitevitch (2010) with well-known English words that varied in *C*—mean response time was ~0.7 s—it is obvious that automatic processes were not engaged in the present task, making the response times uninformative. Therefore, accuracy rate was the only dependent variable of interest. A repeated-measures *ANOVA* was used to analyze the accuracy data with Test and *C* as independent variables. A response was marked as correct if 2 out of the 3 phonemes were produced correctly. This measure of accuracy is more sensitive than a completely correct measure of accuracy (i.e., 3 out of 3 phonemes) and has been used in other studies of word learning [see Storkel et al. (2006) for a discussion regarding the different measures of accuracy].

The results showed that there was no main effect of *C*, but there was a significant main effect for test [*F*_(3, 31)_ = 283.01, *p* < 0.0001]. These main effects, however, must be interpreted in the context of the significant interaction observed between Test and *C*; *F*_(1, 31)_ = 6.17, *p* < 0.001. Planned comparisons showed that there was no significant difference in *C* at Test 1 (High *C*: *Mean* = 0.45, *SD* = 0.18; Low *C*: *Mean* = 0.41, *SD* = 0.20) or at Test 2 (High *C*: *Mean* = 0.69, *SD* = 0.21; Low *C*: *Mean* = 0.71, *SD* = 0.19), all *p* > 0.1. However, a significant difference between accuracy rates of the high (*Mean* = 0.40, *SD* = 0.21) and low *C* nonwords (*Mean* = 0.27, *SD* = 0.17) was observed at Test 3, which occurred after 1 week elapsed and with no additional exposure to the stimuli, *F*_(1, 31)_ = 13.39, *p* < 0.001; *Cohen'sd* = 0.7. Statistical conventions indicate that *Cohen's d* (Cohen, 1988) ~0.2–0.3 is considered a small effect, ~0.5 is considered a medium effect, and greater than 0.8 is considered a large effect. By these conventions, the effect observed in the present experiment is medium to large in magnitude.

Of the responses that were not correct, the most common “error” that was produced was the response “Don't know” (69.3%). The next most common type of “error” (19.9%) was to use one of the nonwords to name the wrong object (e.g., in Test 1 a participant might use the same novel name in response to three different objects, but by Test 3 the correct name-nonobject pairing had been established for the items). Another 6.8% of the errors were responses in which participants would produce the initial phoneme of the nonword, but nothing else of the nonword (much like what happens in the tip-of-the-tongue state with real words). The remaining responses (3.9%) were completely wrong (i.e., the participant created their own nonword to name the nonobject).

## Discussion

In this study novel words with high *C* were learned better than novel words with low *C*, but only after multiple exposures and a 1-week delay between final exposure and final test (i.e., only at Test 3). Numerous studies have shown that delays of ~1-week may be required to fully integrate the representation of a novel word into the lexicon, and for that novel word to affect the processing of one of its neighbors (e.g., Gaskell and Dumay, 2003; Tamminen and Gaskell, 2013). For example, Gaskell and Dumay (2003) report that after an initial exposure, a novel word (e.g., *cathedruke*) facilitates processing of a similar sounding real word (e.g., *cathedral*); similar sounding words that are already established in the lexicon tend to compete with each other. However, after a week without any further exposure the novel word impedes processing of the real word, as measured by performance on the real word in a lexical decision task, suggesting that the novel word has been successfully integrated (i.e., interacting with other established words) in the lexicon. In the present case, learners needed to form a representation of the novel word, and connect that representation to the representations of many well-known neighbors rather than just one word as in the study by Gaskell and Dumay (2003). Thus, it is not surprising that a relatively long period of time may be required for the observed influence of the relationships among all the neighbors of a novel word on the learning/retrieval of that novel word to emerge.

To account for the present results we appeal to: (1) the word-learning framework described in Storkel et al. (2006) and simulated in Vitevitch and Storkel (2013), and (2) the network diffusion framework described in Chan and Vitevitch (2009) and simulated in Vitevitch et al. (2011).

Storkel et al. (2006) suggested that the partial phonological overlap that exists between a novel word and the representations of known words in the lexicon strengthen the newly formed lexical representation of a novel word (see also Jusczyk et al., 1994). A newly formed representation that resembles many known words in the lexicon will be strengthened to a greater extent than a newly formed representation that resembles few known words in the lexicon, leading to the advantage for learning novel words with dense compared to sparse neighborhoods observed by Storkel et al. (2006) and others (e.g., Stamer and Vitevitch, 2012).

However, the novel words in the present study had as neighbors the same number of known words; they instead differed in the extent to which the real word neighbors were neighbors with each other, or *C*. Here we turn to the framework described by Chan and Vitevitch (2009), which started with the network structure for the phonological lexicon observed by Vitevitch (2008), and included the additional assumption that activation would diffuse from an initially activated node to the nodes that it was connected to, and then on to the nodes that they in turn were connected to (which included the node from which activation was initially received). Although other models of cognitive processing often include additional parameters such as inhibition, decay of activation, threshold levels, etc., no such assumptions were made in the description offered by Chan and Vitevitch (2009) and simulated in Vitevitch et al. (2011). That is, Vitevitch et al. (2011) used a much simpler model to account for the observed results.

In the case of a word with low *C* in the mental lexicon, Chan and Vitevitch (2009) suggested that the small number of interconnections among the neighbors would result in some of the activation from the neighbors spreading back to the target word, and the remaining activation dispersing to the rest of the network. In the case of spoken word recognition, which was the process investigated by Chan and Vitevitch (2009), the strongly activated target word would “stand out” from the less activated neighbors, resulting in target words with low *C* being retrieved rapidly and accurately from the lexicon. However, in the case of word-learning, the focus of the present investigation, the “target” word is actually a weak, nascent representation, requiring supplemental activation from its neighbors—as suggested by the work of Storkel et al. (2006)—in order to become a fully integrated lexical representation (i.e., a known word). In a neighborhood with low *C*, activation is dispersed to the rest of the network, leaving little activation to strengthen the representation of the novel word, leading to the difficulty in acquiring word-forms with low *C*.

In the case of a word with high *C* in the mental lexicon, where the neighbors are highly interconnected with each other, Chan and Vitevitch (2009) suggested that most of the activation would remain amongst the interconnected neighbors rather than spread to the rest of the network as happens for words with low *C*. With a highly activated target word as well as highly activated neighbors, discrimination of the target word becomes more difficult, resulting in slower and less accurate retrieval of target words with high *C* from the lexicon. However, in the case of word-learning, the activation that recirculates amongst the neighbors (rather than being dispersed to the rest of the network) is precisely what a nascent representation needs to become a fully integrated lexical representation. In a neighborhood with high *C*, the activation that circulates amongst the neighbors serves to strengthen the representation of the novel word, leading to the ease in acquiring such word-forms (despite the difficulty that such word-forms experience later in the word recognition process). Note that a similar mechanism was proposed by Vitevitch et al. (2012) to account for the influence of *C* on the process of redintegration, which occurs when lexical representations in long-term memory are used to activate degraded lexical representations in short-term memory.

The influence of *C* in various processes—production (Chan and Vitevitch, 2010), recognition (Chan and Vitevitch, 2009), memory (Vitevitch et al., 2012), and now word-learning—hints toward new avenues for future investigation. For example, the picture-naming task used in the present study and in Chan and Vitevitch (2010) may provide researchers with a methodology that can map the transition from a nascent representation to a well-learned word in the lexicon. Recall that Chan and Vitevitch (2010) observed that well-known English words with low *C* were named more quickly than well-known English words with high *C*. In a future study one could continually train and test participants on these novel words until the picture-naming times to those items were comparable to the picture-naming times observed for well-known English words (i.e., the response times drop from ~4 to 0.7 s). At that point, one could then examine if the influence of *C* changed from the learning advantage for high *C* words observed in the present study to the production advantage for low *C* words observed by Chan and Vitevitch (2010).

The influence of *C* on various processes also suggests that characteristics of individual words are not the only things that influence processing. Rather, various language-related processes are also influenced by the relationships that exist among neighbors in the lexicon. Network science offers a wide array of statistical tools to analyze relationships between individual words in the lexicon (i.e., the *micro*-level of the network), characteristics of the over-all structure of the lexicon (i.e., the *macro*-level of the network), as well as relationships that exist at various levels in between (i.e., the *meso*-level of the network). Future studies could examine structural characteristics at other levels of the lexical network (e.g., Siew, 2013; Vitevitch et al., 2014) to determine how they influence word-learning. Even if network science measures are not used in future studies to examine the relationships that exist among words, an increasing amount of evidence is making it clear that more than just the characteristics of the individual word influence various lexical processes.

## Authors note

The experiment in this report partially fulfilled the requirements for a master's degree in psychology awarded to Rutherford Goldstein. Michael S. Vitevitch chaired the thesis. This work was supported in part by NIDCD grant T32—DC00052 Training Researchers in Language Impairments.

### Conflict of interest statement

The authors declare that the research was conducted in the absence of any commercial or financial relationships that could be construed as a potential conflict of interest.
