# Supplementary material for: The influence of clustering coefficient on word-learning: how groups of similar sounding words facilitate acquisition
Source: Front Psychol. 2014 Nov 18;5:1307. doi: 10.3389/fpsyg.2014.01307 (PMC4235275; doi:10.3389/fpsyg.2014.01307)
Supplement: Supplementary file 1 [file DataSheet1.DOCX]

**Appendix A**

| **Nonword** | ***C*** | **Phonotactic Probability** | **Biphone Probability** | **Number of Real Word Neighbors** | **Concreteness Rating** | **Semantic Set Size** | **First Word Associate Strength** | **Second Word Associate Strength** |
| --- | --- | --- | --- | --- | --- | --- | --- | --- |
| faup | 0 | 0.1 | 0.0008 | 1 | 5.6 | 11 | 0.06 | 0.06 |
| boIf | 0.2 | 0.1 | 0.0012 | 3 | 3.8 | 11 | 0.07 | 0.06 |
| fub | 0.2 | 0.11 | 0.0013 | 4 | 5.6 | 11 | 0.11 | 0.09 |
| jog | 0.2 | 0.09 | 0.0022 | 3 | 5.3 | 10 | 0.06 | 0.05 |
| gIf | 0.22 | 0.15 | 0.0044 | 5 | 3.9 | 10 | 0.17 | 0.13 |
| kɛf | 0.25 | 0.17 | 0.0039 | 8 | 5.2 | 11 | 0.1 | 0.09 |
| hɑm | 0.26 | 0.16 | 0.008 | 12 | 3.7 | 11 | 0.09 | 0.06 |
| maɪd | 0.27 | 0.15 | 0.0079 | 20 | 5.1 | 10 | 0.09 | 0.06 |
| mʌn | 0.27 | 0.22 | 0.0192 | 21 | 3.2 | 11 | 0.13 | 0.1 |
| gaɪm | 0.29 | 0.12 | 0.0019 | 8 | 3.7 | 10 | 0.05 | 0.05 |
| pek | 0.30 | 0.17 | 0.0084 | 23 | 4.4 | 11 | 0.13 | 0.06 |
| mɑt | 0.30 | 0.18 | 0.0114 | 22 | 4.4 | 11 | 0.3 | 0.17 |

The Low *C* nonwords used in the experiment (in the International Phonetic Alphabet).

**Appendix B**

The High *C* nonwords used in the experiment (in the International Phonetic Alphabet).

| **Nonword** | ***C*** | **Phonotactic Probability** | **Biphone Probability** | **Number of Real Word Neighbors** | **Concreteness Rating** | **Semantic Set Size** | **First Word Associate Strength** | **Second Word Associate Strength** | |
| --- | --- | --- | --- | --- | --- | --- | --- | --- | --- |
| jɑr | 0.41 | 0.16 | 0.0225 | 12 | 4.6 | 10 | 0.18 | 0.16 |  |
| gin | 0.41 | 0.18 | 0.0044 | 11 | 4.5 | 10 | 0.2 | 0.1 |  |
| fep | 0.43 | 0.13 | 0.0035 | 6 | 5.4 | 10 | 0.15 | 0.1 |  |
| pæb | 0.43 | 0.17 | 0.0083 | 12 | 5.2 | 11 | 0.1 | 0.09 |  |
| gɛk | 0.49 | 0.17 | 0.0061 | 8 | 3.2 | 10 | 0.11 | 0.09 |  |
| daub | 0.49 | 0.09 | 0.0012 | 3 | 3.6 | 10 | 0.13 | 0.12 |  |
| meb | 0.58 | 0.12 | 0.0062 | 8 | 4.3 | 11 | 0.17 | 0.16 |  |
| dug | 0.62 | 0.11 | 0.0023 | 10 | 3.4 | 11 | 0.17 | 0.11 |  |
| jek | 0.64 | 0.12 | 0.0052 | 13 | 3.4 | 10 | 0.1 | 0.09 |  |
| noIb | 0.67 | 0.05 | 0.0006 | 2 | 5.2 | 10 | 0.32 | 0.07 |  |
| pib | 0.68 | 0.13 | 0.0048 | 8 | 4.3 | 10 | 0.3 | 0.13 |  |
| wib | 0.75 | 0.10 | 0.0035 | 5 | 5.9 | 11 | 0.13 | 0.06 |  |
